# Supplementary material for: A Cross-Sectional Study: Systematic Quantification of Chemerin in Human Cerebrospinal Fluid
Source: Biomedicines. 2024 Nov 1;12(11):2508. doi: 10.3390/biomedicines12112508 (PMC11592017; doi:10.3390/biomedicines12112508)
Supplement: Supplementary file 1 [file biomedicines-12-02508-s001.zip › biomedicines-3224902-supplementary.pdf]

Supplementary

Supplementary table S1: The proportion of patients in BMI subgroups did not differ significantly between male and female

	Male (n = 161)	Female (n = 229)
BMI < 25 kg/m² (n = 161)	59	102
BMI ≥ 25 kg/m² (n = 229)	102	127

The proportion of patients in BMI subgroups did not differ significantly between male and female. Chi-Quadrat-Test was applied for calculation of p values and statistical significance ($p < 0.05$). BMI: body mass index; CRP: C-reactive protein.

Supplementary table S2: Correlation analyses of chemerin levels in serum (A) and CSF (B)

Total cohort (n = 390) Parameter	p	*rho*
**A) Correlation analysis of serum chemerin levels with:**		
Anthropometric parameters		
Age [y]	< 0.001	+0.238
Weight [kg]	< 0.001	+0.258
Height [cm]	0.033	-0.108
BMI [kg/m²]	< 0.001	+0.358
Lipoprotein-metabolism		
Triglycerides [mg/dL]	0.006	+0.311
Inflammation		
CRP [mg/L]	< 0.001	+0.500
Leukocytes [giga/L]	< 0.001	+0.178
Serum parameters		
Total albumin [g/L]	< 0.001	-0.285
GOT [U/L]	< 0.001	+0.186
GPT [U/L]	< 0.001	+0.178
Bilirubin [mg/dL]	< 0.001	-0.299
Urea [mg/dL]	< 0.001	+0.194
LDH [U/L]	< 0.001	+0.212
CSF parameters		
Cell count [µL]	0.005	-0.142
IgA [g/L]	0.042	+0.129
Adipokine		
Chemerin in CSF [ng/mL]	< 0.001	+0.245
**B) Correlation analysis of CSF chemerin levels with:**		
Anthropometric parameters		
Age [y]	< 0.001	+0.367
Weight [kg]	0.001	+0.164
Height [cm]	0.004	+0.147
BMI [kg/m²]	0.035	+0.107
Carbohydrate-metabolism		
Plasma glucose [mg/dL]	0.029	+0.116
Lipoprotein-metabolism		
LDL cholesterol [mg/dL]	0.034	+0.264
Inflammation		
CRP [mg/L]	0.024	+0.115
Serum parameters		
GOT [U/L]	< 0.001	+0.215
GPT [U/L]	< 0.001	+0.199
Creatinine [mg/dL]	< 0.001	+0.217

Urea [mg/dL]	< 0.001	+0.209
LDH [U/L]	< 0.001	+0.215
IgG [g/L]	0.016	-0.123
IgM [g/L]	0.025	-0.142
CSF parameters		
Cell count [µL]	< 0.001	-0.176
Total protein [g/L]	< 0.001	+0.262
Albumin [g/L]	< 0.001	+0.305
IgG [g/L]	0.003	+0.153
IgA [g/L]	0.002	+0.195
CSF/serum Albumin ratio	< 0.001	+0.301

Correlation analyses of chemerin levels in serum (A) and CSF (B). Chemerin levels were quantified by ELISA. The Spearman-rho test was applied for calculation of p values and statistical significance ($p < 0.05$). BMI: body mass index; LDL: low-density ; CRP: C-reactive protein; CSF: cerebrospinal fluid; GOT: glutamic oxaloacetic transaminase; GPT: glutamic-pyruvic transaminase; LDH: lactate dehydrogenase; Ig: immunoglobulin.

Supplementary table S3: Exclusion of confounding variables of significant correlations found in patients without indications of neurological disease

Confounding variable	Chemerin serum [ng/mL] vs. BMI [kg/m^2]
age [y]	Rho: +0.378; p < 0.001; DG: 167
CRP [mg/L]	Rho: +0.399; p < 0.001; DG: 163
male (n = 58)	Rho: 0.061; p = 0.648
female (n = 110)	Rho: + 0.577; p < 0.001
	Chemerin serum [ng/mL] vs. CRP [mg/L]
Age [y]	Rho: +0.342; p < 0.001; DG: 163
BMI [kg/m^2]	Rho: +0.317; p < 0.001; DG: 163
male (n= 56)	Rho: +0.512; p < 0.001
female (n=110)	Rho: +0.612; p < 0.001
	Chemerin Serum vs. CSF levels [ng/mL]
age [y]	Rho: +0.159; p = 0.038; DG: 167
BMI [kg/m^2]	Rho: +0.207; p = 0.007; DG: 167
CRP [mg/L]	Rho: +0.209; p = 0.007; DG: 163
male (n=58)	Rho: − 0.073; p = 0.589
female (n=110)	Rho: + 0.343; p < 0.001
	Chemerin CSF [ng/mL] vs. Age [y]
BMI [kg/m^2]	Rho: +0.376; p < 0.001; DG: 167
CRP [mg/L]	Rho: +0.361; p < 0.001; DG: 163
male (n=58)	Rho: + 0.396; p = 0.002
female (n=112)	Rho: + 0.464; p < 0.001
	Chemerin CSF [ng/mL] vs. CSF/serum albumin ratio
age [y]	Rho: +0.260; p < 0.001; DG: 161
BMI [kg/m^2]	Rho: +0.381; p < 0.001; DG: 161
CRP [mg/L]	Rho: +0.391; p < 0.001; DG: 157
male (n=56)	Rho: + 0.122; p = 0.372
female (n=108)	Rho: + 0.505; p < 0.001
	CSF/serum Chemerin ratio vs. CSF/serum albumin ratio
age [y]	Rho: +0.206; p = 0.008; DG: 161
BMI [kg/m^2]	Rho: +0.258; p < 0.001; DG: 161

CRP [mg/L]	Rho: +0.244; p = 0.002; DG: 157
male (n=56)	Rho: + 0.186; p = 0.170
female (n=108)	Rho: + 0.126; p = 0.194

Exclusion of confounding variables of significant correlations found in patients without indications of neurological disease: Partial correlation analyses was perfomed on correlation analyses in Figure 6 to exclude confounding variables. CRP: C-reactive protein; BMI: body mass index; CSF: cerebrospinal fluidDG: Degree of freedom

Supplementary table S4: Correlation analyses of chemerin serum (A) and CSF (B) levels in patients without neurological diseases

Control group (n=170) Parameter	p	*rho*
**A) Correlation analysis of serum chemerin levels with:**		
Anthropometric parameters		
Age [y]	< 0.001	+0.258
Weight [kg]	< 0.001	+0.304
Height [cm]	0.033	-0.163
BMI [kg/m^2]	< 0.001	+0.414
Inflammation		
CRP [mg/L]	< 0.001	+0.570
Leukocytes [giga/L]	< 0.001	+0.302
Serum parameters		
Total albumin [g/L]	< 0.001	-0.420
GOT [U/L]	0.009	+0.201
GPT [U/L]	0.003	+0.230
Bilirubin [mg/dL]	< 0.001	-0.374
Urea [mg/dL]	< 0.001	+0.254
LDH [U/L]	0.018	+0.184
Adipokine		
Chemerin in CSF [ng/mL]	0.008	+0.201
**B) Correlation analysis of CSF chemerin levels with:**		
Anthropometric parameters		
Age [y]	< 0.001	+0.427
Weight [kg]	0.014	+0.187
BMI [kg/m^2]	0.022	+0.176
Carbohydrate-metabolism		
Plasma glucose [mg/dL]	0.030	+0.176
Serum parameters		
GPT [U/L]	0.006	+0.213
Urea [mg/dL]	0.005	+0.215
LDH [U/L]	0.019	+0.183
IgG [g/L]	0.041	-0.160
CSF parameters		
Total protein [g/L]	< 0.001	+0.335
Albumin [g/L]	< 0.001	+0.384
Lactate [mmol/L]	< 0.001	+0.261
IgG [g/L]	< 0.001	+0.268
IgA [g/L]	0.004	+0.287
CSF/serum Albumin ratio	< 0.001	+0.381

Correlation analyses of chemerin serum (A) and CSF (B) levels in patients without neurological diseases. Chemerin levels were quantified by ELISA. The Spearman-

rho test was applied for calculation of p values and statistical significance ($p < 0.05$). BMI: body mass index; CRP: C-reactive protein; CSF: cerebrospinal fluid; GOT: glutamic oxaloacetic transaminase; GPT: glutamic-pyruvic transaminase; LDH: lactate dehydrogenase; Ig: immunoglobulin.

Supplementary table S5: Study characteristics of sub cohorts of patients suffering from MS, ID, epilepsy, CVD or PC and matched controls

A)	MS (n=68)	Matched controls (n=68)
Sex m/f (%)	23/45 (33.8/66.2)	23/45 (33.8/66.2)
Age [y]	35.57 ± 11.12 [19-59]	35.46 ± 11.91 [18-62]
BMI <25/≥25kg/m² (%)	30/38 (44.1/55.9)	31/37 (45.6/54.4)
B)	ID (n=30)	Matched controls (n=30)
Sex m/f (%)	13/17 (43.3/56.7)	13/17 (43.3/56.7)
Age [y]	55.53 ± 17.02 [22-81]	55.97 ± 17.18 [21-87]
BMI <25/≥25kg/m² (%)	13/17 (43.3/56.7)	13/17 (43.3/56.7)
C)	Epilepsy (n=50)	Matched controls (n=50)
Sex m/f (%)	34/16 (68.0/32.0)	34/16 (68.0/32.0)
Age [y]	49.68 ± 19.46 [18-84]	48.74 ± 19.93 [17-83]
BMI <25/≥25kg/m² (%)	22/28 (44.0/56.0)	29/21 (58.0/42.0)
D)	CVD (n=46)	Matched controls (n=46)
Sex m/f (%)	28/18 (60.9/39.1)	28/18 (60.9/39.1)
Age [y]	59.67 ± 14.87 [22-84]	57.98 ± 15.92 [22-87]
BMI <25/≥25kg/m² (%)	15/31 (32.6/67.4)	24/22 (52.2/47.8)
Chemerin serum [ng/mL]	395.96 ± 154.77 [205.12-864.27]	370.21 ± 127.19 [191.23-775.28]
Chemerin CSF [ng/mL]	35.06 ± 10.87 [9.83-61.58]	33.68 ± 9.27 [13.07-51.25]
Chemerin CSF/serum ratio x10^{-3}	97.27 ± 41.79 [37.20-230.01]	99.64 ± 39.42 [39.82-193.04]
E)	PC (n=26)	Matched controls (n=26)
Sex m/f (%)	5/21 (19.2/80.8)	5/21 (19.2/80.8)
Age [y]	40.04 ± 13.23 [20-68]	39.85 ± 12.44 [18-65]
BMI <25/≥25kg/m² (%)	1/25 (3.8/96.2)	0/26 (0/100)
Chemerin serum [ng/mL]	385.18 ± 99.60 [211.94-568.17]	395.87 ± 103.17 [210.19-599.64]
Chemerin CSF [ng/mL]	29.77 ± 8.49 [15.98-46.26]	30.11 ± 7.44 [14.17-43.61]
Chemerin CSF/serum ratio x10^{-3}	81.90-27.93 [29.67-123.68]	80.85 ± 28.05 [27.79-160.93]

Study characteristics of sub cohorts of patients suffering from MS, ID, epilepsy, CVD or PC and matched controls. Anthropometric and chemerin levels in serum and CSF in MS (A), ID (B), epilepsy (C), CVD (D) or PC (E) patients and matched controls are displayed. Mean values, standard deviation and range are displayed. Absolute numbers and percentages are shown for classified variables. MS: multiple sclerosis; ID: infectious disease; CVD: cerebrovascular disease; PC: pseudotumor cerebri; BMI: body mass index; CSF: cerebrospinal fluid. P value was calculated by Mann-Whitney-U-Test, a p value of < 0.05 was considered significant.
